# Physical activity delays accumulation of immunosuppressive myeloid-derived suppressor cells

**DOI:** 10.1371/journal.pone.0234548

**Published:** 2020-06-15

**Authors:** Jacob Garritson, Luke Krynski, Lea Haverbeck, James M. Haughian, Nicholas A. Pullen, Reid Hayward

**Affiliations:** 1 School of Sport and Exercise Science and the University of Northern Colorado Cancer Rehabilitation Institute, University of Northern Colorado, Greeley, CO, United States of America; 2 School of Biological Sciences, University of Northern Colorado, Greeley, CO, United States of America; University of Memphis, UNITED STATES

## Abstract

**Background:**

Myeloid-derived suppressor cells (MDSCs) are potent suppressors of immune function and may play a key role in the development and progression of metastatic cancers. Aerobic exercise has been shown to have anticancer effects, yet the mechanisms behind this protection are largely unknown. Therefore, we examined the effects of physical activity on MDSC accumulation and function.

**Methods:**

Female BALB/c mice were assigned to one of two primary groups: sedentary tumor (SED+TUM) or wheel run tumor (WR+TUM). After 6 weeks of voluntary wheel running, all animals were randomly subdivided into 4 different timepoint groups; 16, 20, 24, and 28 days post-tumor injection. All mice were inoculated with 4T1 mammary carcinoma cells in the mammary fat pad and WR groups continued to run for the specified time post-injection. Spleen, blood, and tumor samples were analyzed using flow cytometry to assess proportions of MDSCs.

**Results:**

Cells expressing MDSC biomarkers were detected in the spleen, blood, and tumor beginning at d16. However, since there was no evidence of immunosuppressive function until d28, we refer to them as immature myeloid cells (IMCs). Compared to SED+TUM, levels of IMCs in the spleen were significantly lower (*p* < 0.05) in WR+TUM at day 16 (33.0 ± 5.2%; 23.1 ± 10.2% of total cells, respectively) and day 20 (33.9 ± 8.1%; 24.3 ± 5.1% of total cells, respectively). Additionally, there were fewer circulating IMCs in WR+TUM at day 16 and MDSC levels were significantly lower (*p* < 0.05) in the tumor at day 28 in WR+TUM. Additionally, a non-significant 62% and 26% reduction in metastatic lung nodules was observed at days 24 and 28, respectively. At day 28, MDSCs harvested from SED+TUM significantly suppressed CD3^+^CD4^+^ T cell proliferation (3.2 ± 1.3 proliferation index) while proliferation in WR+TUM MDSC co-cultures (5.1 ± 1.7 proliferation index) was not different from controls.

**Conclusions:**

These findings suggest that physical activity may delay the accumulation of immunosuppressive MDSCs providing a broader window of opportunity for interventions with immunotherapies.

## Introduction

Myeloid-Derived Suppressor cells (MDSCs) are a heterogeneous population of immature immune cells that expand in response to cancer and various other pathological conditions. Originally identified as “natural-suppressor” cells, the MDSC label was later suggested to encompass the myeloid origin and potent immune-suppressive activity of these cells [1,2]. MDSCs are detectable in most cancer patients and perversely function to neutralize antitumor immunity by directly inhibiting the activation and proliferation of CD4^+^ T helper and CD8^+^ Cytotoxic T cells [3]. Additionally, MDSCs appear to increase T cell tolerance of malignant tumor cells and are broadly viewed as one of the primary factors limiting the efficacy of some immunotherapy treatments [4–6]. Thus, any interventive strategy that minimizes the negative influence of MDSCs may significantly improve outcomes for patients treated with immunotherapies.

Two primary subtypes of MDSCs have been characterized in humans and mice, granulocytic polymorphonuclear MDSCs (PMN-MDSCs) and monocytic MDSCs (M-MDSCs) [7]. In mice, both populations of MDSCs express the surface markers CD11b and Gr1, where Gr1 is comprised of the molecules Ly6G and Ly6C. PMN-MDSCs are defined phenotypically as CD11b^+^Ly6G^+^Ly6C^lo^ and M-MDSCs are defined as CD11b^+^Ly6G^–^Ly6C^hi^ [7]. Malignant cancer cells can disrupt normal myelopoiesis and increase production of MDSCs from the bone marrow by secreting systemic growth factors, pro-inflammatory cytokines, and signaling lipids [3]. For example, the cytokine granulocyte-macrophage colony-stimulating factor (GM-CSF) is necessary for the preferential expansion of MDSCs with potent immune-suppressive function [8,9]. Also, interleukin-6 (IL-6) and interleukin-1β (IL-1β) are pro-inflammatory cytokines that have been implicated as drivers of the accumulation of MDSCs in tumors and secondary lymphoid organs [10,11]. Modeling this tumor-dependent MDSC expansion has frequently relied on spontaneous or syngeneic transplantable tumors in immune-intact mice, both of which can lead to the pronounced expansion of both PMN- and M-MDSC cell populations detectable in the bone marrow, spleen and general circulation [12].

Epidemiological evidence suggests that moderate to vigorous physical activity reduces the risk for developing several types of cancer. For instance, considerable evidence indicates that regular physical activity is beneficial and may reduce the risk for developing breast cancer by as much as 30% in a dose-dependent manner, an effect that appears to be independent of confounding factors such as body mass index [13,14]. While the underlying biological mechanisms behind this protection are still largely unknown, several hypotheses have been proposed including reductions in sex hormone levels through improved body composition, lower fasting insulin levels due to increased insulin sensitivity, and beneficial adaptations to chronic low-grade inflammation in trained individuals [15]. Additionally, several studies in animal models have shown that voluntary wheel running can slow the growth rate of transplantable tumors [16–19]. Conclusions from these animal studies suggest that physical activity may improve antitumor immunity by normalizing intratumoral vascularization or acutely mobilizing highly cytotoxic natural killer (NK) cells and T cells to the tumor microenvironment. The role of physical activity on macrophage polarization and function has been explored previously, and it is possible that many of the studies reviewed here [20] inadvertently studied the function of MDSCs due the heterogeneity of these cells. However, to our knowledge, only one recent study has specifically examined the effects of physical activity on MDSCs in a pre-clinical tumor model [21]. Therefore, the aim of this investigation was to determine if voluntary wheel running temporally delays the accumulation of immunosuppressive MDSCs in a murine model of metastatic breast cancer. We hypothesized that mice who engage in physical activity would have reduced MDSC burden in the spleen, blood, and tumor when compared to sedentary control animals. Additionally, we hypothesized that MDSCs purified from physically active mice would be less suppressive toward T cell proliferation *in vitro*.

## Materials and methods

### Animals and wheel running protocol

The study protocol was approved by the Institutional Animal Care and Use Committee (IACUC) of the University of Northern Colorado (protocol number: 1906CE-RH-RM-22). Isoflurane was used to sedate mice during tumor injection. Mice were under sedation for less than 3 minutes during this time. All dissections were performed following lethal injection of heparinized (500 U) sodium pentobarbital (50mg/kg), and all efforts were made to minimize suffering. Female BALB/c mice, 8 weeks of age, were housed individually in a temperature-controlled facility with a 12:12 hour light-dark cycle. Mice were fed standard chow and distilled water *ad libitum*. Mice were randomly assigned to either a wheel run (WR+TUM) or sedentary (SED+TUM) group on day one of the intervention. SED+TUM was restricted to normal cage activity for the duration of the study. WR+TUM animals were given 24-hour access to commercially available running wheels (11.5 cm by diameter) (MiniMitter; Bend, OR) for the duration of the study. Running distance was monitored using a Vital View data acquisition system (MiniMitter; Bend, OR) and running distances were calculated as (3.14 x 11.5 cm • number of revolutions)/(100 cm/m • 1000 m/km) [22]. On week 6 of the study, animals were sedated with isoflurane and inoculated subcutaneously with 1x10^4^ 4T1 cells in the fourth mammary gland on the right side using a 25-gauge syringe needle. Following tumor inoculation, SED+TUM and WR+TUM were randomly subdivided: 12 days (n = 3), 16 days (n = 9), 20 days (n = 9 SED+TUM; 10 WR+TUM), 24 days (n = 9), or 28 days (n = 18). WR+TUM groups continued to run post-injection and subgroups of mice were sacrificed at the specified time points. Additionally, non-tumor control mice, 8 weeks of age, were randomly assigned to a sedentary control (SED) or wheel run control (WR) group (n = 6/group) to confirm 4T1-dependent expansion of MDSCs. WR mice were given free access to cage-mounted running wheels for 10 weeks and SED mice were restricted to normal cage activity. Sample size calculations using an α of 0.05, β of 0.2, and power of 0.8 revealed that a sample size of 4 in non-tumor controls was adequate to detect differences in spleen and blood MDSCs.

### Cell culture, tumor inoculation, and measurement

The syngeneic 4T1 mouse mammary carcinoma (ATCC, CRL-2539) cell line was used to grow orthotopic tumors in the mammary gland. This cell line was originally isolated in 1978 from mammary tumors in BALB/c mice [23]. The 4T1 line was selected because of previous work showing that these tumors significantly expand the proportion of MDSCs detectable in the spleens of tumor-bearing animals [12]. Cells were grown in complete RPMI (cRPMI) medium supplemented with 1% pyruvate, 1% penicillin/streptomycin, 1% HEPES, 1% L-glutamine, 2-Mercaptoethanol, 10% fetal bovine serum (FBS), and maintained in a humidified incubator at 37°C and 5% CO_2_.

On week 6 of the study, TUM groups were sedated with isoflurane and inoculated with 1x10^4^ 4T1 cells in the fourth mammary gland on the right side. Tumor volume, body weight and body condition were monitored three times per week for the duration of the study. Tumor dimensions were measured 3 times per week using digital calipers and tumor volume was estimated using the following equation: π/6 × width × length^2^, where length equals the largest dimension, and width is measured 90° from length. If the dorsal pelvic bone and vertebral column became readily visible and palpable for two consecutive assessments, the percent loss of body mass exceeded 25% of starting mass, or if tumors became ulcerated, the animal was euthanized. If these humane endpoints were reached, the animal was euthanized within one hour of the body condition assessment.

### Tissue preparation and flow cytometric analysis

At the time of sacrifice, animals were euthanized via lethal injection using heparinized (500 U) sodium pentobarbital (50mg/kg). Spleen, blood, and tumor samples were collected and prepared for analysis via flow cytometry. Spleens were mechanically dissociated and filtered through a 100 μm cell strainer. Blood samples were collected via cardiac puncture into a syringe containing 0.1 mL heparin. Tumors were excised and mechanically homogenized using a cell dissociation sieve tissue grinder (Sigma Aldrich), then enzymatically digested in type I collagenase (2 mg/mL; Worthington) for 45 minutes at 37°C on a platform rocker. Following digestion, tumor samples were filtered through a 100 μm cell strainer. Single cell suspensions of splenocytes, tumor, and peripheral blood were cleared of red blood cells by incubating in ammonium-chloride-potassium lysis buffer for 3 min at room temperature.

Cleared cells were resuspended in PBS-1% bovine serum albumin (BSA) buffer and Fc receptors blocked by incubating samples on ice for 10 min in anti-mouse CD16/32 antibody (BioLegend). Cells were then resuspended in antibody solution containing fluorochrome-conjugated monoclonal antibodies against CD11b, Ly-6G, Ly-6C, CD8, CD4, CD335, or the appropriate isotype control (BioLegend). The final concentration for all antibodies used was 1:100 per million cells in 100 μl of PBS-1% BSA. Samples were incubated on ice in the dark for 45 min and analyzed on an Attune NxT flow cytometer (ThermoFisher). Initial forward scatter/side scatter gating was used to exclude debris and doublets from analysis. Unstained and isotype controls were used to exclude background fluorescence and non-specific binding. PMN-MDSCs were defined as CD11b^+^Ly6G^+^Ly6C^lo^ and M-MDSCs were defined as CD11b^+^Ly6G^–^Ly6C^hi^ [3]. Lymphocyte populations included in the analysis were CD4^+^ T cells, CD8^+^ T cells, and CD335^+^ NK cells [24].

### MDSC purification using Fluorescent activated cell sorting

PMN-MDSCs and M-MDSCs were purified from the spleens of tumor bearing mice for functional assessment using fluorescence activated cell sorting (FACS). Single cell suspensions of splenocytes were cleared of red blood cells and stained for CD11b, Ly-6G, and Ly-6C as described above. Gates were established to identify CD11b^+^Ly6G^+^Ly6C^lo^ and CD11b^+^Ly6G^-^Ly6C^hi^ cells for sorting. These subpopulations of MDSCs were pooled and sorted into the same 15 mL conical containing cRPMI supplemented with 20% FBS using a Sony SH800S cell sorter. After sorting, cells were centrifuged for 7 min at 300g. The cell pellet was resuspended in cRPMI growth medium at a concentration of 1x10^6^/mL and set aside.

### T cell suppression assay

#### Antibody coating of microwell plates

Anti-CD3ε antibody (BioLegend) was prepared in sterile PBS (5 μg/mL) and 50 μL was dispensed into each experimental well of a 96-well flat-bottom tissue culture plate; 50 μL of sterile PBS was added to unstimulated control wells. The plate was tightly covered with Parafilm and incubated for 2 hours at 37°C. Before adding cells, antibody solution was removed, and each well was washed 2 times with sterile PBS.

#### Addition of cells

All samples were run in duplicate. Splenocytes were harvested from a naïve female Balb/c mouse, 12–15 weeks of age, and cleared of red blood cells as described above. The cell suspension was resuspended in cRPMI at a concentration of 2x10^6^/mL and centrifuged for 7 min at 300g. The splenocytes were resuspended in 5μM CellTrace Violet (1:1000) (ThermoFisher) and incubated at 37°C for 20 min protected from light. CellTrace Violet was quenched by adding cRPMI and incubating splenocytes for an additional 5 min at 37°C before pelleting and resuspending in cRPMI. Next, 100 μL of the splenocyte cell suspension (2x10^5^ total cells) was added to each well of the antibody coated plate from above. The MDSC suspension (100 μL; 1x10^5^ total cells) was added to each experimental well for a 2:1 splenocyte to suppressor ratio. To account for higher cell numbers and added competition for resources in the experimental wells, 3x10^5^ splenocytes were added to control wells to equal the total number of cells in experimental wells. cRPMI (100 μL) was added to unstimulated control and stimulated control wells for a final volume of 200 μL/well. Next, soluble anti-CD28 (2 μg/mL) antibody (BioLegend) was added to each stimulated well to promote T cell proliferation. Finally, samples were incubated at 37°C for 72 h and duplicate samples were pooled, stained with anti-CD3, CD4, and CD8 antibody then analyzed on an Attune NxT flow cytometer. Proliferation analysis based on CellTrace intensity was performed using FCS express 6 (DeNovo Software).

### Lung metastasis

Animals were euthanized as described above before an incision was made along the midline of the abdomen through the ribcage and up toward the salivary glands. The trachea was identified and elevated by threading a 200 μL pipette tip underneath. Using a 27-G syringe needle, India Ink (10% India Ink and 0.1% ammonium hydroxide) was injected into the lungs via the trachea until resistance was felt. The lungs were removed and rinsed briefly with deionized water then transferred to a glass vial containing 3 mL of Fekete’s solution (50% ethanol, 6% formaldehyde, 3% glacial acetic acid). After at least 10 min, surface metastatic nodules were visualized as white dots on black lungs. Lungs were transferred to a dish and individual metastatic lesions were manually counted on the surface of both the right and left lung and an average nodule count was obtained for each group [25]. Group averages were compared to determine the effects of physical activity on the number of metastatic lung nodules.

### Statistical analysis

Statistical analyses were performed using GraphPad Prism Software 8 (La Jolla, CA). Group averages are reported as mean ± standard error of the mean. Significant differences between two groups were determined using Students *t*-test or *t*-test with Welch’s correction if variances were unequal; comparisons between more than two groups were performed using one-way ANOVA, repeated measures one-way ANOVA, or two-way ANOVA as appropriate. P-values with *p* < 0.05 were considered significant.

## Results

### 4T1 tumors disrupt normal myelopoiesis

**Fig 1:**
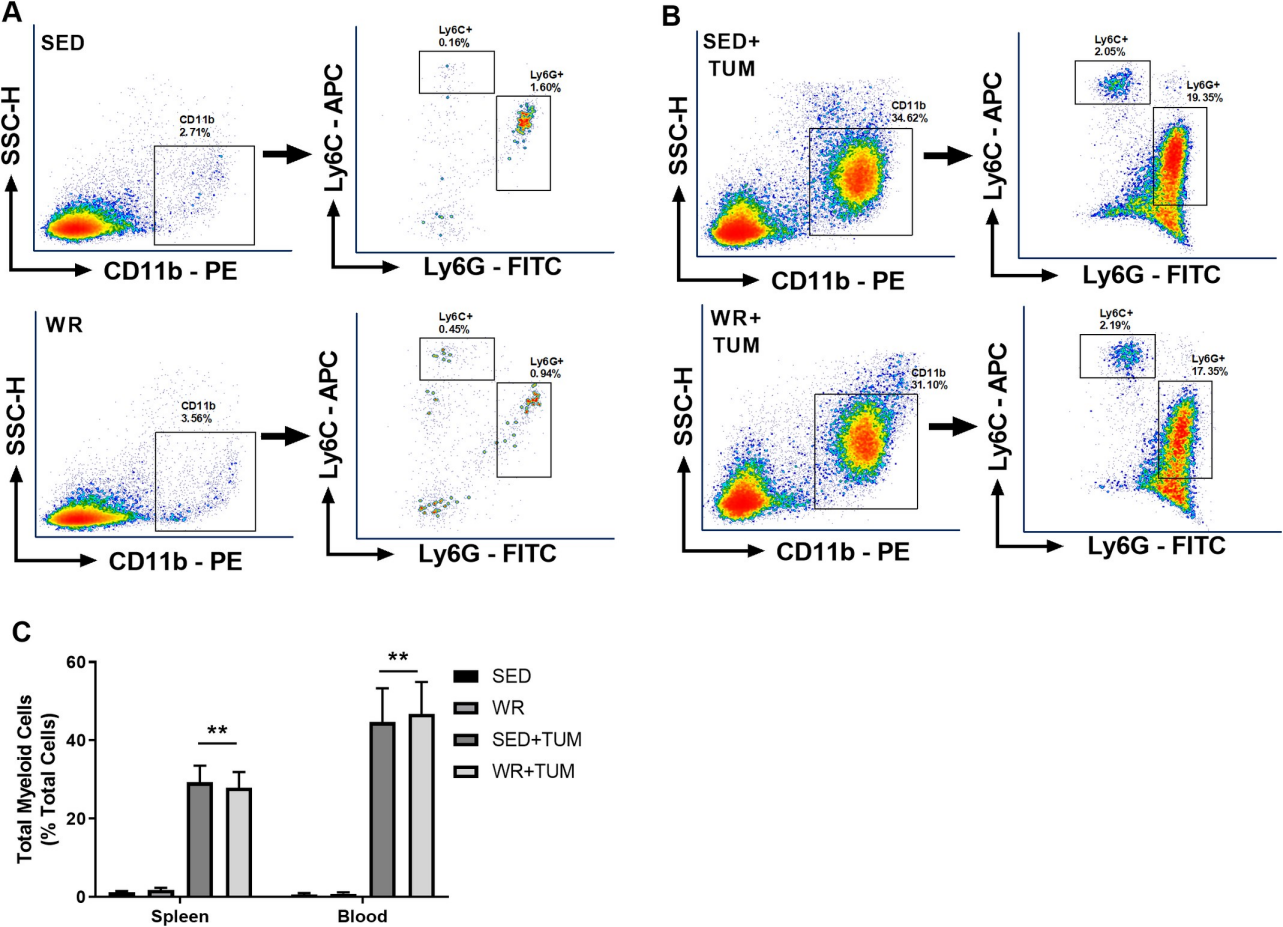
4T1 tumors stimulate CD11b^+^Ly6G^+^Ly6C^lo^ and CD11b^+^Ly6G^-^Ly6C^hi^ cell production. (A-B) Representative flow cytometry density plots from the spleen of individual day 28 non-tumor or tumor-bearing mice demonstrating a profound uptick of myeloid cells in tumor-bearing mice. Following doublet exclusion, primary gates were established for CD11b^+^ cells. Secondary gates were established for Ly6G^+^ cells and Ly6C^+^ cells to identify different subsets of myeloid cells. (C) CD11b^+^Ly6G^+^Ly6C^lo^ and CD11b^+^Ly6G^-^Ly6C^hi^ cells were combined and reported as total myeloid cells in the spleen and blood. Means ± SEM (n = 6/group for non-tumor groups; n = 18/group for tumor-bearing groups). **p < 0.01 vs. WR and SED non-tumor bearing mice. For spleen and blood MDSCs, a one-way ANOVA with Tukey multiple comparisons was used.

To confirm that 4T1 tumors disrupt normal myelopoiesis, spleen and blood samples from tumor-bearing mice and non-tumor controls were analyzed using flow cytometry. The gating strategy for identification of CD11b^+^Ly6G^+^Ly6C^lo^ and CD11b^+^Ly6G^-^Ly6C^hi^ Immature myeloid cells (IMCs)/MDSCs is presented in Fig 1A. Physical activity did not change these individual subpopulations of cells, so they were combined and reported as the total proportion of myeloid cells in a sample. After flow cytometric analysis, a significant accumulation of total myeloid cells (p < 0.05, F_(3, 44)_ = 9.38) was observed in the spleens of both WR+TUM and SED+TUM compared to WR and SED (Fig 1C), indicating a tumor-dependent disruption of myelopoiesis, which is characteristic of the 4T1 tumor model [11]. Similarly, circulating blood myeloid cells in WR+TUM and SED+TUM were significantly higher (p < 0.01, F_(3, 44)_ = 6.16) than WR and SED controls. Further, myeloid levels were not different between WR and SED (Fig 1C) indicating that physical activity does not significantly alter myelopoiesis in tumor-free animals.

### Physical activity delays the accumulation of IMCs/MDSCs

**Fig 2:**
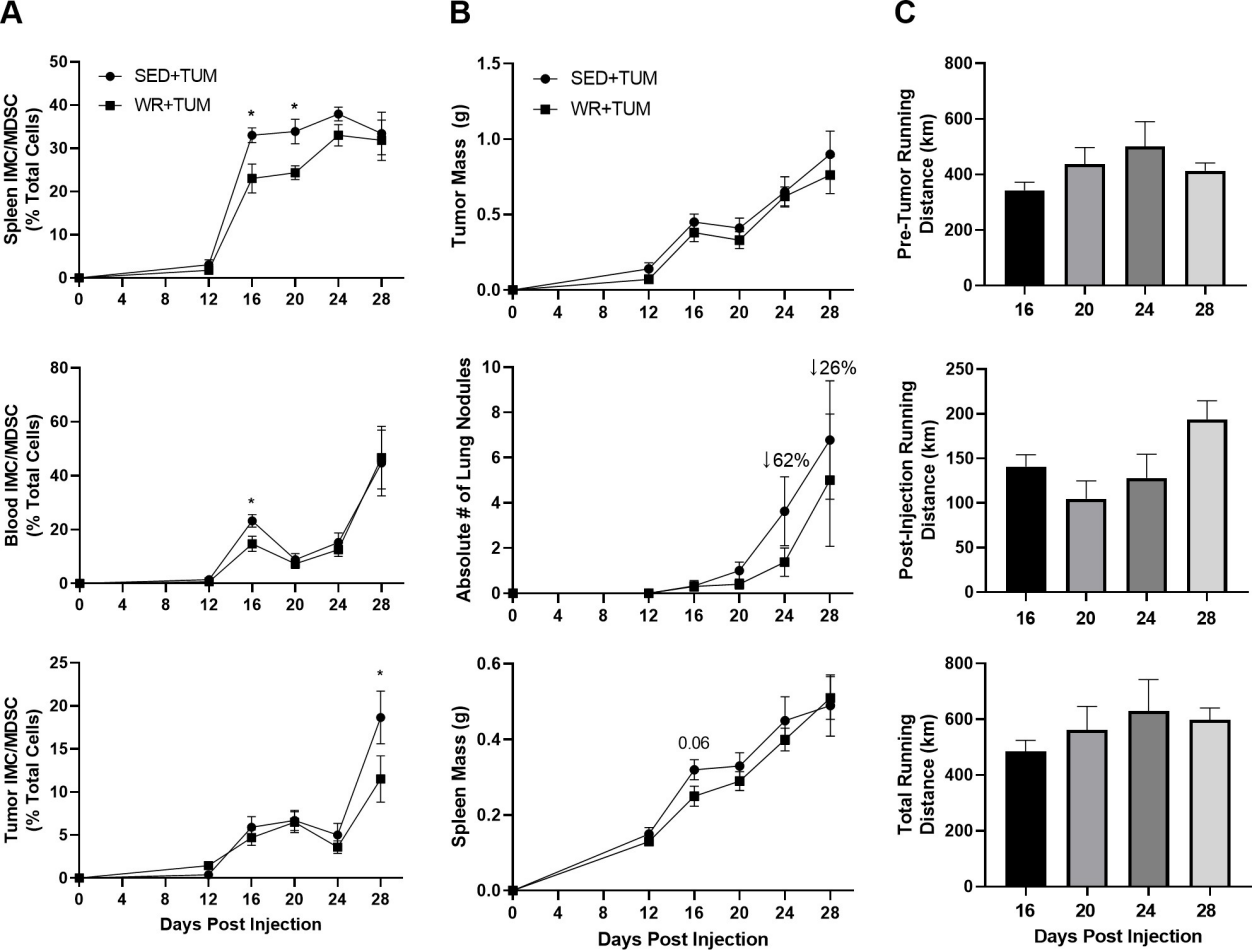
IMC/MDSC-related tumor progression and metastasis. (A) Total accumulation of IMCs/MDSCs in the spleen (top), blood (middle), and tumor (bottom) at the specified time-points post-4T1 cell injection. (B) Tumor mass (top), metastatic lung nodules (middle), and spleen mass (bottom) over the course of the study. (C) Pre-tumor running distance (top), post-4T1 cell injection running distance (middle), and total running distance (bottom) in WR+TUM animals. Means ± SEM (n = 9-18/group per timepoint; n = 3/group for day 12 timepoint). *p < 0.05 vs. WR+TUM. For tissue IMCs/MDSCs, tumor mass, lung nodules, and spleen mass, a student’s t test was used to compare groups. For running distances, a one-way ANOVA was used to compare different timepoint groups.

**Fig 3:**
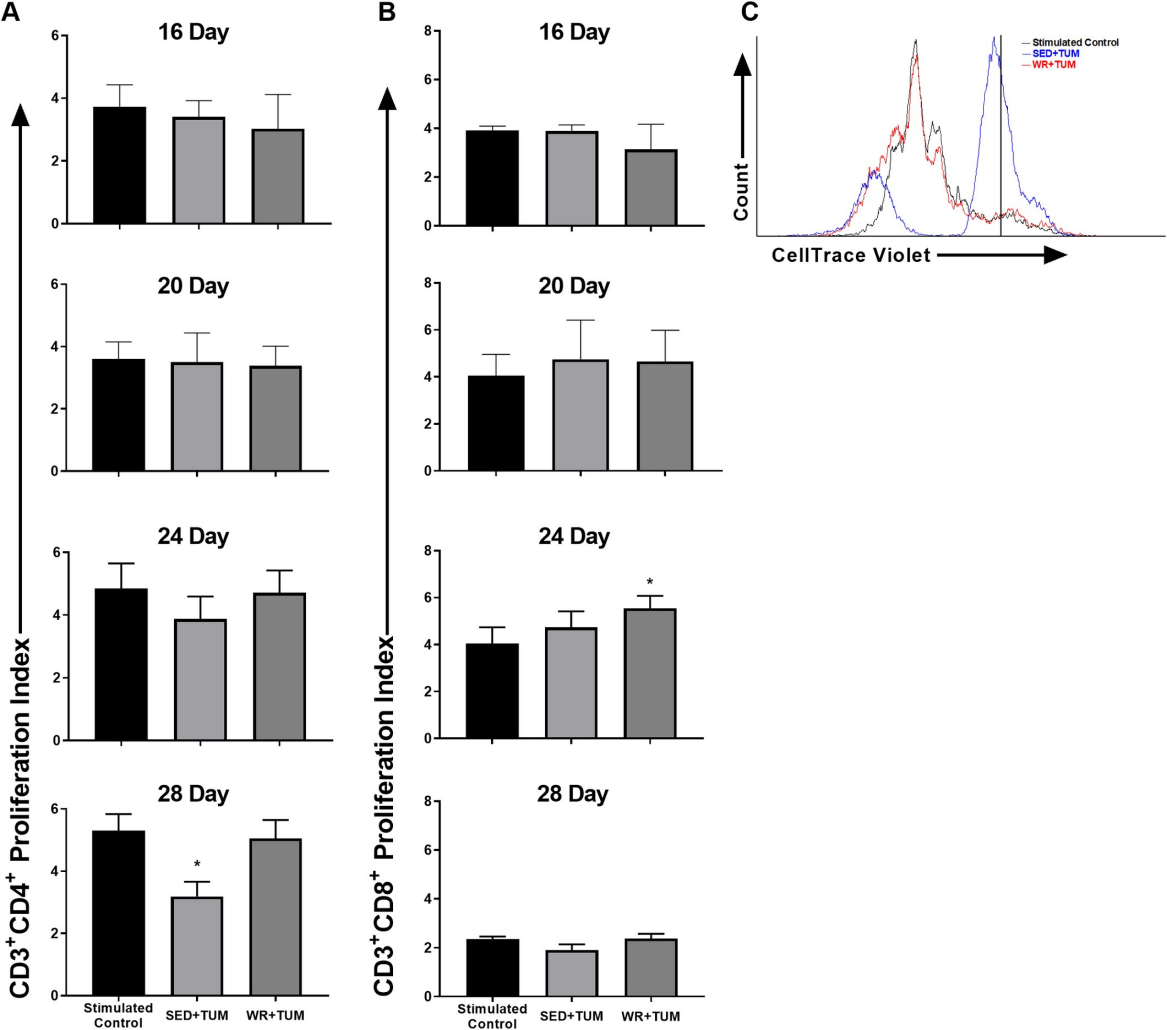
T cell suppression assay in MDSC co-cultures. (A) CD3^+^CD4^+^ T cell proliferation in stimulated controls and IMC/MDSC co-cultures at the specified timepoint post-4T1 cell injection. (B) CD3^+^CD8^+^ T cell proliferation in stimulated controls and IMC/MDSC co-cultures at the specified timepoint post-4T1 cell injection. (C) Representative CD3^+^CD4^+^ T cell proliferation plot from day 28 samples. All samples were run in duplicate and median CellTrace Violet fluorescence in unstimulated samples was used to set the starting undivided population (vertical black line). Splenocytes were stimulated to proliferate using anti-CD3ε and anti-CD28 antibody. Means ± SEM (n = 8-10/group). *p < 0.05 vs. stimulated control. For statistical analysis a repeated-measures ANOVA with Tukey’s multiple comparison was used.

Spleen, blood, and tumor samples were analyzed in tumor-bearing mice at the indicated time points to determine the effects of physical activity on MDSC accumulation. As later experiments demonstrate (Fig 3), these cells were not functionally suppressive toward T cell proliferation until day 28, thus by functional definition they were labeled IMCs. By 12 days after injection, palpable tumors were present (Fig 2B); however, IMCs were not accumulating in the spleen, blood, or tumor (Fig 2A). At day 16, a marked increase in the number of these cells was observed in tumor bearing animals. In WR+TUM animals, IMCs in the spleen were significantly (*p* < 0.05) lower than SED+TUM at day 16 (23.0 ± 10.0% vs. 33.0 ± 5.2%) and day 20 (24.3 ± 5.1% vs. 33.9 ± 8.0%) (Fig 2A). The effect of physical activity on IMC/MDSC accumulation in the spleen was lost by day 24 and 28 as no differences were observed between groups. Additionally, at day 16, circulating IMCs in the blood were significantly lower (*p* < 0.05) in WR+TUM (14.7 ± 8.5%) when compared to SED+TUM (23.2 ± 7.1%) (Fig 2A). A recent study showed that a combination of physical activity and energy restriction reduced total MDSCs in the tumor microenvironment (TME) 35 days post-tumor implantation by about 15% [21]. In comparison, we observed that MDSCs in the TME of WR+TUM (11.5 ± 8.1%) were significantly lower (*p* < 0.05) than SED+TUM (18.7 ± 9.2%) at day 28 independent of energy restriction. While the delay in accumulation of IMCs/MDSCs did not translate to smaller tumors at any timepoint (Fig 2B), there was a non-significant 62% and 26% reduction in metastatic lung nodules in WR+TUM at days 24 and 28, respectively (Fig 2B). Spleen mass progressively increased as tumor size increased, and no differences were observed with physical activity. Additionally, no differences were observed in the running distance between different timepoint groups (Fig 2C). This is expected as mice tend to run more during the first weeks of being housed in a wheel running cage and progressively decrease their running volume each subsequent week, resulting in only slightly higher post-injection distances in the 24 and 28 day groups [26].

### Physical activity alters the suppressive function of MDSCs

Myeloid cells were identified as described in Fig 1B and purified using FACS. Average purity of sorted samples was > 80%, which is within the normal expected range when using this method [27]. Representative dot plots of pre and post-sort samples are provided in S1 Fig. Pathological activation of MDSCs has been described previously as a gradual process that is related to tumor progression [28]. Our results support this idea as a significant accumulation of myeloid cells with the typical MDSC surface marker expression phenotype was observed at earlier timepoints (Fig 2A), however these cells did not become significantly suppressive toward T cell proliferation until day 28. Therefore, MDSCs at earlier timepoints are labeled IMCs rather than *bona fide* MDSCs. At day 28, CD3^+^CD4^+^ T cell proliferation in SED+TUM MDSC co-cultures (3.19 ± 0.47) was significantly lower (*p* < 0.05, F_(2, 12)_ = 5.6) than stimulated control samples (5.30 ± 0.53) (Fig 3A). In comparison, CD3^+^CD4^+^ cell proliferation in WR+TUM MDSC co-cultures (5.05 ± 0.59) was not significantly suppressed suggesting that physical activity delayed the acquisition of suppressive function by MDSCs. At all timepoints, CD3^+^CD8^+^ T cell proliferation was not significantly suppressed in either group (Fig 3B). However, CD3^+^CD8^+^ T cell proliferation in WR+TUM co-cultures (5.54 ± 0.53) at day 24 was significantly higher (*p* < 0.05, F_(8, 16)_ = 17.0) than stimulated controls (4.04 ± 0.69) suggesting that CD3^+^CD8^+^ T cell proliferation was increased in WR+TUM co-cultures at day 24.

### Effects of 4T1 tumors on tissue resident lymphocytes

**Fig 4:**
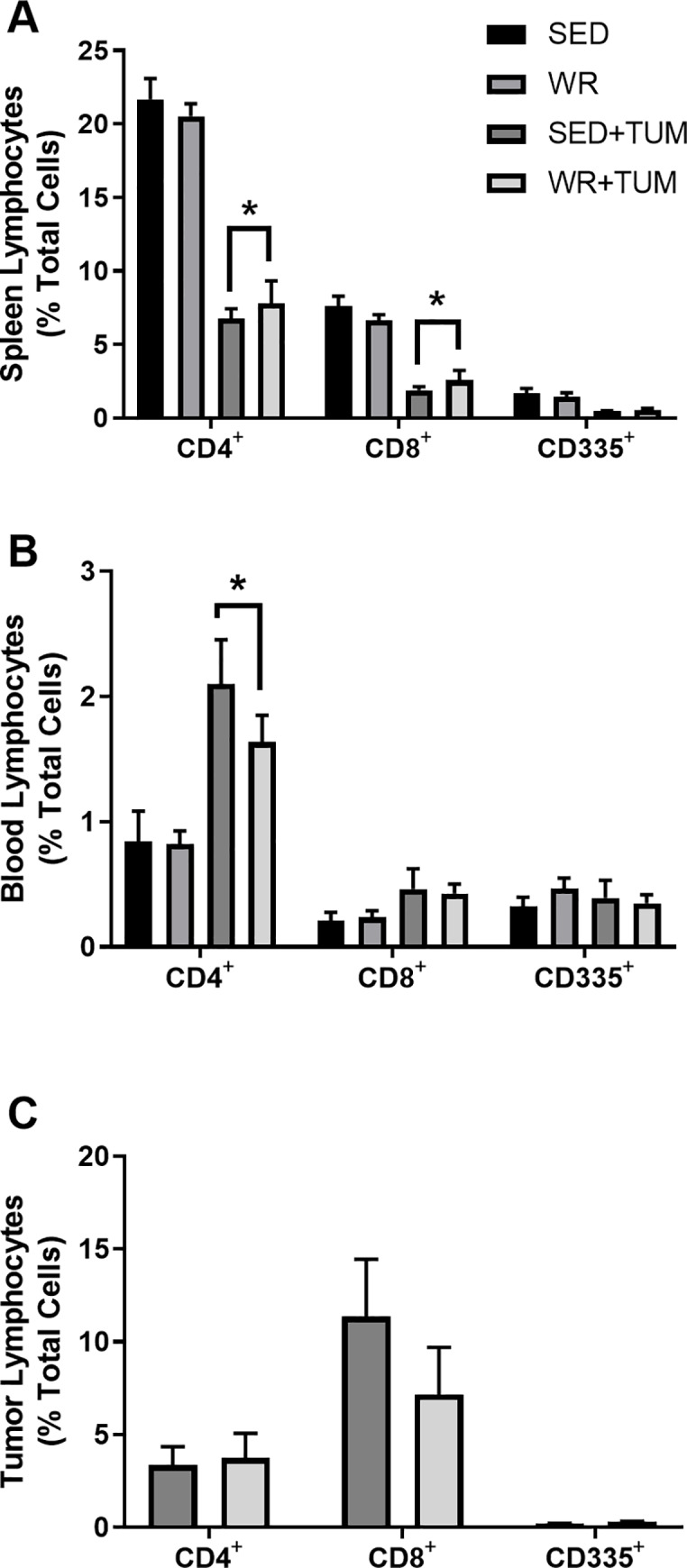
Tissue resident and circulating T cell (CD4^+^, CD8^+^), and NK cell (CD335^+^) lymphocytes. (A) Spleen, (B) blood, and (C) tumor samples were analyzed at 28 days post-4T1 cell injection. *p < 0.05 vs. SED and WR control animals. Data are presented as means ± SEM (n = 6-9/group). Spleen and blood lymphocytes were analyzed using a two-way ANOVA with Tukey multiple comparison. Tumor Lymphocytes were analyzed using a Student’s t-test.

Consistent with systemic immune suppression, the presence of 4T1 tumors significantly reduced CD4^+^ and CD8^+^ (*p* < 0.05, F_(3, 67)_ = 59.2) lymphocyte populations in the spleens of tumor-bearing mice when compared to WR and SED controls (Fig 4A). Additionally, tumor-bearing mice had significantly higher proportions of CD4^+^ (*p* < 0.05, F_(3, 49)_ = 2.30) cells in the blood (Fig 4B). Paradoxically, higher numbers of CD4^+^ cells have been correlated with disease progression in 4T1 tumor-bearing mice [29]. No differences were observed between WR+TUM and SED+TUM in the tumor (*p* > 0.05) for any other lymphocyte populations analyzed, including CD335^+^ NK cells (Fig 4C) which have been shown to be altered by physical activity in this location in other murine tumor models [17].

## Discussion

A growing body of literature suggests that physical activity, both acute and chronic, can favorably modulate immune function, with beneficial effects that potentially extend into the prevention and treatment of cancer. Physical activity and exercise is known to modulate inflammatory signaling, as well as the expansion and recruitment of both myeloid and lymphoid lineage immune cells [30]. Nearly all leukocyte populations are mobilized to the blood during acute exercise and moderate intensity exercise is known to improve chemotaxis, phagocytosis, and oxidative burst activity in neutrophils [31]. MDSCs are immature myeloid cells routinely associated with a poorer prognosis in several cancer types [7] and pharmacological blockade of MDSCs can slow the growth of transplantable tumors [32,33]. To date, only one study has investigated the effects of voluntary wheel running on the accumulation of MDSCs in a pre-clinical animal model and the present study aimed to determine if physical activity could improve antitumor immunity by reducing the accumulation of IMCs/MDSCs in an orthotopic mouse model of breast cancer. Our data show that voluntary wheel running significantly delayed the accumulation of IMCs/MDSCs in the spleen, blood, and tumor, however, these effects did not translate to smaller tumors by size and weight. Few groups have investigated the effects of physical activity on metastasis in 4T1 tumor-bearing mice and here we observed a non-significant 62% and 26% reduction in the number of metastatic lung nodules at days 24 and 28, respectively. In comparison, a recent study showed that wheel running in 4T1 tumor-bearing mice increased metastatic burden in the lungs when mice began running concurrently with tumor injection [34]. Turbitt et al. recently reported that energy restriction preventing weight gain in combination with voluntary wheel running significantly reduced M- and PMN-MDSCs in the spleen and tumor which lead to a decrease in tumor growth. They also observed a non-significant decrease in metastatic burden in the lungs in wheel running mice fed *ad libitum* [21]. By comparison, we report that physical activity can reduce the numbers of these cell populations independent of energy restriction. Taken together, these results suggest that weight management may be important to maximize the beneficial immunomodulatory effects of physical activity in tumor-bearing mice. One potential mechanism explaining these findings is the normalization of tumor vasculature with physical activity. For example, others have shown that 18 days of wheel running following implantation of 4T1 tumor cells resulted in improved vessel maturity, perfusion, and reduced hypoxia in the tumor microenvironment [35]. Reductions in hypoxia have been shown to reduce the accumulation of MDSCs in 4T1 tumors, decrease the expression of PD-L1, and increase the recruitment of T cells to the tumor microenvironment [36].

A hallmark of MDSCs is the ability to suppress T cell proliferation. MDSCs purified from SED+TUM mice appeared to preferentially suppress CD3^+^CD4^+^ T cell proliferation while MDSCs from WR+TUM mice did not significantly suppress T cell proliferation at 28 days, suggesting that physical activity may help normalize myelopoiesis and consequently the function of MDSCs. Compared to the findings of Turbitt et al. where they observed an increased proliferation of CD4^+^ T cells harvested from physically active tumor-bearing mice [21], we showed that physical activity may directly alter the ability of MDSCs to suppress naïve T cells evidenced by reduced T cell suppression in MDSC co-cultures at day 28. Additionally, CD3^+^CD8^+^ T cell proliferation was significantly higher than controls in WR+TUM IMC co-cultures at day 24 suggesting cytotoxic T cell proliferation was increased. MDSCs are often described as immature immune cells that are highly plastic depending on the context of the microenvironment. While it is beyond the scope of this paper, it is possible that the anti-inflammatory effects of physical activity may help normalize the aberrant myelopoiesis associated with accumulation and activation of MDSCs.

Physical activity did not influence the proportions of CD335^+^ NK cells in any of the tissues that were analyzed. This was unexpected considering previous work in melanoma, liver and lung cancers where catecholamines released during wheel-running activity was associated with increased NK cell populations and diminished tumor growth [16]. This discrepancy could be tumor cell line-dependent as only a minor, non-significant increase in NK cells was observed with 4T1 tumors, and perhaps the robust expansion of MDSCs in the 4T1 breast tumor model supersedes an effective NK cell response [37]. Additionally, tumor-bearing mice had significantly higher proportions of CD4^+^ cells in the blood compared to non-tumor controls Huang et al. reported that CD4^+^ cells are significantly increased in the blood of 4T1 tumor bearing animals with advancing tumor stages and that these cells progressively change their differentiation status from anti-tumor Th1’s to pro-tumor T regulatory (Treg) cells and Th17 cells [29]. Th17 cells for example have been implicated in MDSC accumulation through production of interleukin 17 (IL-17) [38] and endurance exercise has been shown to reduce Th17 cytokine production *in vitro* [39]. A recent report showed that physical activity in 4T1 tumor-bearing mice increased the tumor-CD8^+^/FoxP3^+^ ratio when compared to sedentary mice [40], however, due to our limited phenotypical characterization of CD4^+^ cells, future work is needed to confirm whether physical activity protects against an increase of pro-tumor Tregs and Th17 cells in the tumor and circulation.

Physical activity had no effect on the rate of tumor progression in our study. Mixed results have been reported in the literature, with some studies in 4T1 tumor bearing mice showing that voluntary wheel running can slow the growth of 4T1 tumors [16,22,40], while other recent reports show that wheel running alone had no effect on tumor growth in 4T1 tumor-bearing mice [21,34]. We propose that pre-training is important for the observed antitumor effects of physical activity which may be related to the running habits of rodents. Mice run the highest volumes the first several weeks after being housed with a running wheel and progressively decrease their running volume during subsequent weeks [26]. Adaptations that promote an anti-inflammatory environment and progression toward more low-moderate running intensity may be important for the anticancer effects observed in pre-training models. In comparison, animals that begin running on the day of tumor inoculation may run at a volume and intensity that is ultimately immunosuppressive, negating any potential anticancer effects.

Immune checkpoints regulate the immune system by promoting self-tolerance and preventing autoimmunity. Tumors are known to co-opt these immune checkpoint pathways to evade immune surveillance by T cells specific for tumor antigens. Checkpoint inhibitor immunotherapy targets these immune checkpoints by blocking the interaction between checkpoint proteins that inactivate tumor-specific T cells [41]. High levels of circulating MDSCs have been used as a predictor of the clinical response to immunotherapy with higher MDSCs being associated with treatment failure [3,42]. In patients treated with the checkpoint inhibitor ipilimumab, low levels of circulating MDSCs were associated with increased survival and improved treatment outcomes [43]. Additionally, depletion of MDSCs in pre-clinical mouse models significantly improves the outcomes of immunotherapy [44] and exercise has been shown to regulate inflammatory signals such as IL-6 and IL-1β that are implicated in MDSC accumulation [10,31,45]. Also, human breast cancer survivors who participated in a six month endurance training intervention had significant reductions in serum levels of systemic inflammatory cytokines such as TNF-α and IL-6 [46]. Further, an acute bout of exercise has been shown to increase serum concentrations of the anti-inflammatory cytokines interleukin 10 (IL-10) and interleukin-1 receptor antagonist (IL-1ra) [47]. Taken together, the anti-inflammatory effects of exercise described above in both animal models and human subjects have the potential to regulate several of the mechanisms involved in the accumulation and maintenance of MDSCs, specifically the IL-1β and IL-6 signaling pathways. However, future work is necessary to determine whether these factors underly the beneficial effects of physical activity observed in this study.

In conclusion, the present study investigated the effects of voluntary wheel running on the accumulation of pro-tumor IMC/MDSCs and anti-tumor lymphocytes in a murine model of metastatic breast cancer. Despite a profound uptick in IMC/MDSC production in tumor-bearing animals, voluntary wheel running was able to delay the accumulation of immunosuppressive MDSCs. Furthermore, MDSCs harvested from physically active mice were less suppressive toward T cell proliferation *in vitro* and IMCs enhanced cytotoxic T cell proliferation at day 24 post-tumor injection. Thus, findings from the present study suggest that physical activity may be a cost-effective way to reduce the severity of aberrant myelopoiesis that results in the accumulation of pathologically activated MDSCs, which could potentially improve the efficacy of now common immunotherapies such as PD-1/PD-L1 checkpoint inhibitors.

## Limitations

While the 4T1 tumor model has been used extensively to study MDSC biology, there are some limitations to our design. It is possible that with a higher ratio of MDSCs, some suppressive effects may be observed at earlier timepoints, however, due to low numbers of MDSCs at the early timepoints, only a 2:1 splenocyte-to-suppressor ratio was used. Additionally, different subpopulations of MDSCs exert their immune suppressive effects using different mechanisms. Again, due to low cell numbers at early timepoints, especially in the M-MDSC population, both populations were pooled together in this study making it difficult to determine where the observed effects of physical activity are occurring. These findings were limited to the 4T1 breast cancer tumor model and future research into the effects of physical activity on MDSC accumulation in additional tumor models will help to confirm the results presented here. Experiments using S100A9 knockout mice, which have a reduced capacity to generate MDSCs, would help confirm that the observed effects in this study were physical activity-dependent. Additional assays further characterizing the phenotype and function of T cells are necessary to determine the effects of physical activity on antitumor immunity. Finally, mice were individually housed to accurately measure running activity. This may potentially affect immune function, as mice experience less stress in social groups and have an increased metabolic rate to maintain core temperature when housed individually [48]. All animals in the present study were housed individually to account for these effects.

## Supporting information

S1 Fig Representative Day 28 spleen samples before and after MDSC purification using FACS. (A) Day 28 spleen sample from an individual WR+TUM animal before sorting and (B) after sorting.
(TIF)

S2 Fig (PPTX)

S3 Fig (TIF)
