# Supplementary figures and images for: Physical activity delays accumulation of immunosuppressive myeloid-derived suppressor cells
Source: PLoS One. 2020 Jun 15;15(6):e0234548. doi: 10.1371/journal.pone.0234548 (PMC7295224; doi:10.1371/journal.pone.0234548)

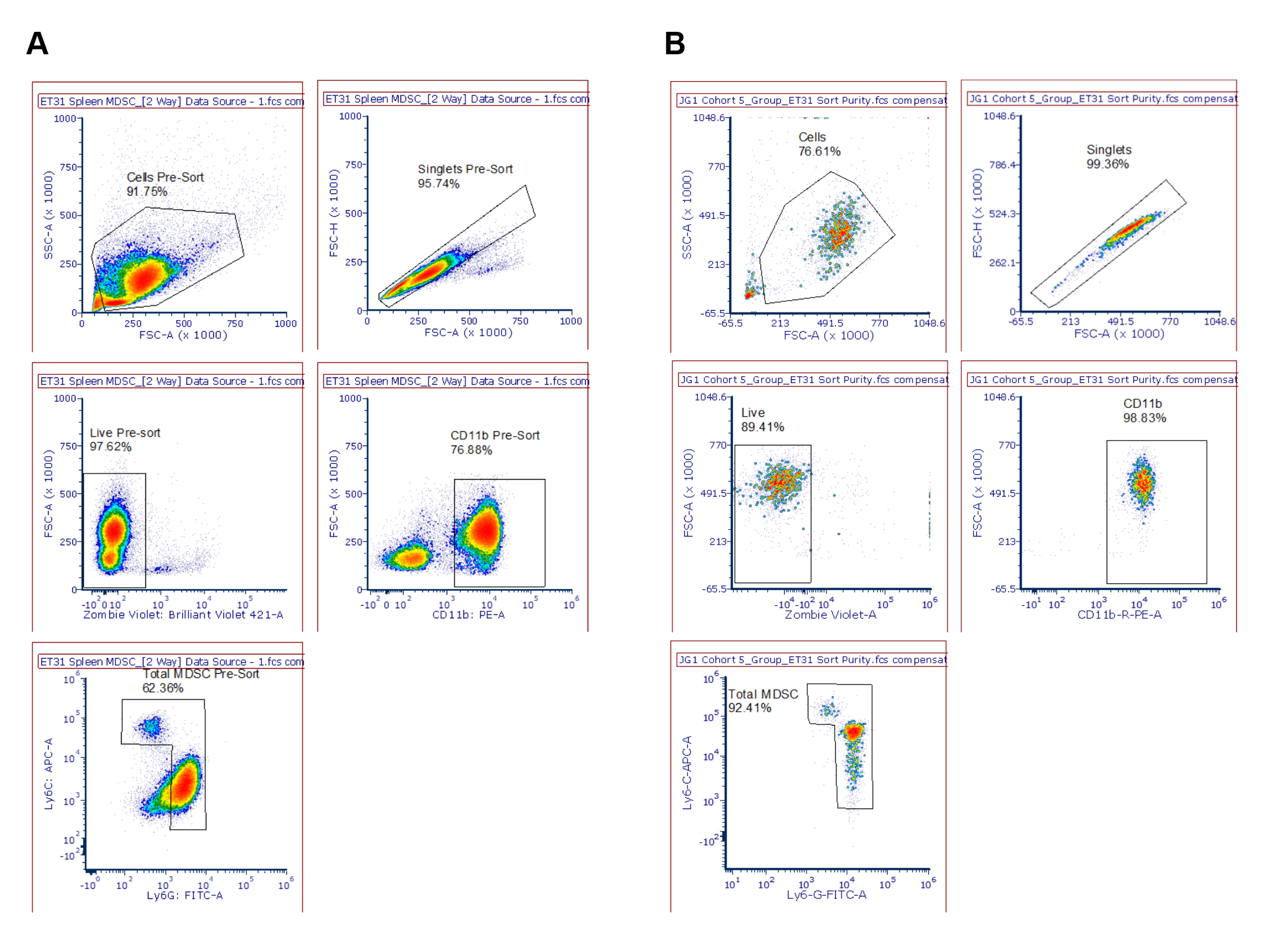

Supplement: S1 Fig — (A) Day 28 spleen sample from an individual WR+TUM animal before sorting and (B) after sorting. (TIF) [file pone.0234548.s001.tif]

## Slide 1
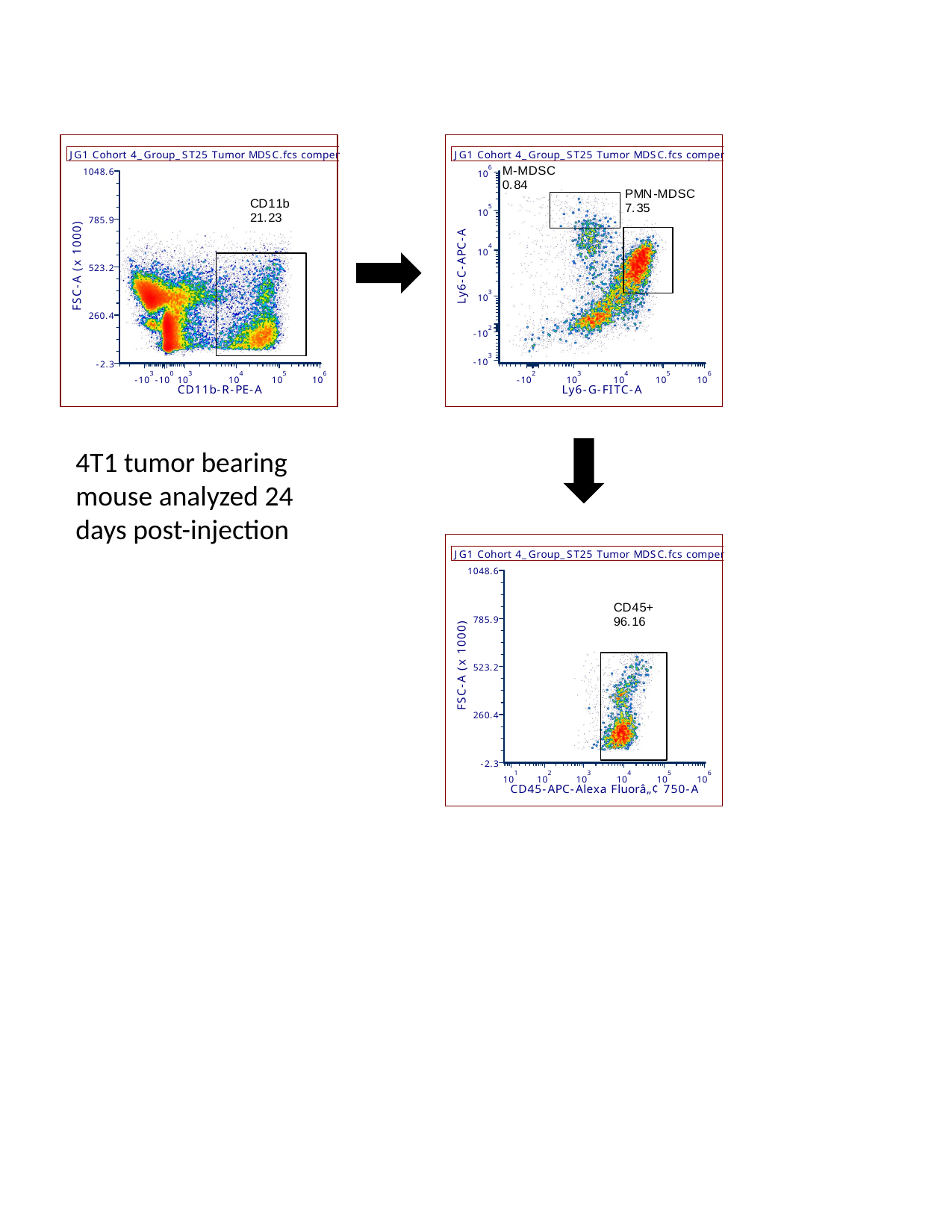

4T1 tumor bearing mouse analyzed 24 days post-injection

Supplement: S2 Fig — (PPTX) [file pone.0234548.s002.pptx]

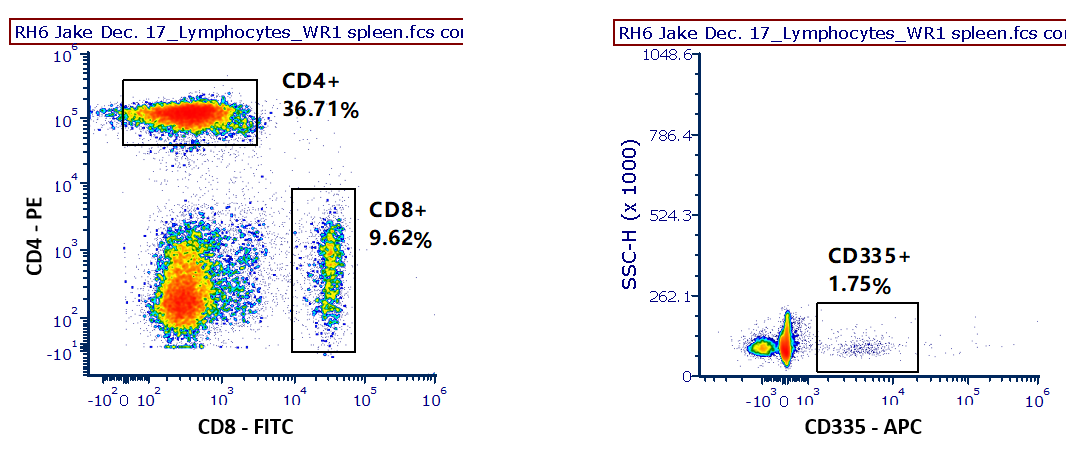

Supplement: S3 Fig — (TIF) [file pone.0234548.s003.tif]
